# Diagnosis of the phase function of random media from light reflectance

**DOI:** 10.1038/srep22535

**Published:** 2016-03-03

**Authors:** Min Xu

**Affiliations:** 1 Physics Department, Fairfield University, CT 06824, USA

## Abstract

Light reflectance has been widely used to diagnose random media in both *in situ* and *in vivo* applications. The quantification of the phase function of the medium from reflectance measurements, however, remains elusive due to the lack of an explicit connection between the light reflectance profile and the phase function. Here we first present an analytical model for reflectance of scattered light at an arbitrary source-detector separation by forward-peaked scattering media such as biological tissue and cells. The model incorporates the improved small-angle scattering approximation (SAA) to radiative transfer for sub-diffusive light reflectance and expresses the dependence of the light reflectance on the phase function of the scattering medium in a closed form. A spreading length scale, *l*_Θ_, is found to characterise subdiffusive light reflectance at the high spatial frequency (close separation) limit. After validation by Monte Carlo simulations, we then demonstrate the application of the model in accurate determination of the complete set of optical properties and the phase function of a turbid medium from the profile of subdiffusive and diffusive light reflectance.

Elastic scattering of light has long been used to diagnose the random medium. Reflectance spectroscopy and imaging is a widely used noninvasive method to measure the optical properties of random media such as atmosphere, ocean, and tissue, including the absorption coefficient (*μ*_*a*_) and the reduced scattering coefficient 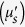. These parameters provide valuable information regarding the microarchitecture and biochemical composition of the medium and has been applied in, for example, cloud remote sensing^1^, monitoring cell apoptosis^2^, skin characterisation^3^, and cancer detection^4^. The quantification of the phase function of the medium, which describes the angular distribution of the scattered light upon single interaction with the medium and contains the ultimate information about the microenvironment of the medium, from reflectance measurements, however, remains elusive. Reflectance of scattered light is inherently a difficult problem as light propagation in a random medium is governed by radiative transfer (RT)^5^ and the commonly adopted diffusion approximation to RT breaks down at a short source-detector separation^6^. There is a tremendous need for an accurate analytical model of reflectance at an arbitrary source-detector separation, amenable for rapid quantitative assessment of optical properties and in particular, the phase function of a random medium. Some empirical models for reflectance at a close source-detector separation were proposed recently with limited applicable domains^7^,^8^. As the phase function of the scattering medium impacts significantly sub-diffusive reflectance at a close source-detector separation, an analytical model which can relate explicitly sub-diffusive reflectance to the phase function and can recover the complete set of optical parameters (including the phase function) of the random media from the reflectance profile is hence greatly desirable.

In the present work, we report here an analytical model for reflectance of scattered light at an arbitrary source-detector separation by forward-peaked scattering media and its application in diagnosis of the phase function of such media. The model incorporates the small-angle scattering approximation (SAA) to radiative transfer for sub-diffusive light reflectance at a close source-detector separation and expresses the dependence of the light reflectance on the phase function of the scattering medium in a closed form. The performance of the model is then verified by Monte Carlo simulations. The application of the proposed model in diagnosis of random medium is demonstrated at the end with an emphasis on determining the phase function of the scattering medium.

## Results and Discussion

Consider light reflectance of a collimated beam incident along the direction 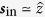 and backscattered in the direction 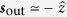 by a forward-peaked scattering medium with the interface at *z* = 0. In such media, the non-diffuse photons have only encountered a small number of large angle scattering and will be classified into the *n*th order non-diffuse photons by the number, *n*, of large angle scattering. The leading contribution is due to the first order non-diffuse photons which have experienced multiple small angle scattering and *exactly* one large angle scattering and can be described in a closed form using the small angle scattering approximation to radiative transfer^9^. The first order non-diffuse photons will be called SAA photons. The second order non-diffuse photons (“snake” photons) dominate in coherent backscattering (CBS) as SAA photons are commonly suppressed in CBS measurements in the circular polarisation preserved channel^10^,^11^.

**Figure 1:**
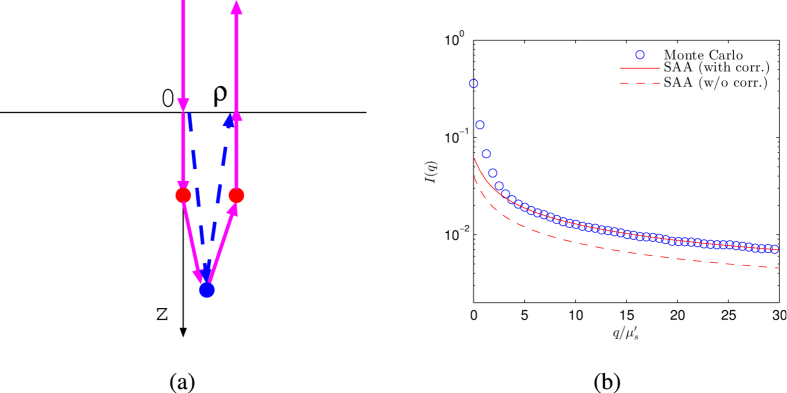
(**a**) Backscattering of the first order non-diffuse photons which encounter multiple small-angle scattering (“red” scatterers) and one single large angle scattering (“blue” scatterer). The photon may take the isotropic (dashed lines) vs forward scattering (solid lines) route with the probability of *p*_iso_ and 1 − *p*_iso_, respectively, at the first or last scattering event. (**b**) The inclusion of *S*^eff′^ improves the accuracy of SAA. The comparison to Monte Carlo simulations for light reflectance from polystyrene sphere suspensions of diameter 1.5 *μm* in water is shown. The wavelength of the incident light is 0.515 *μm*.

Backscattering of SAA photons is mainly determined by the spread of the scattering angles in the forward directions and the backscattering efficiency. The phase function *p*(*θ*) (normalised as 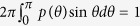) of the scattering medium is assumed to split into a forward-peaked component and an isotropic scattering one, i.e., *p*_SAA_(*θ*) = (1 − 2*p*_*b*_)*p*_Forward_(*θ*) + (2*π*)^−1^*p*_*b*_ 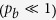. The SAA spread function for a collimated beam in the direction ***s***_0_ incident at the origin (***r*** = 0) on a stratified medium to reach the depth *z* is given by^12^:


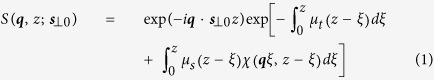


where ***q*** is the spatial frequency on the *xy* plane, **s**_⊥0_ is the projection of **s**_0_ on the interface, *μ*_*t*_ ≡ *μ*_*s*_ + *μ*_*a*_ with *μ*_*s*_ being the scattering coefficient, and *χ*(***v***, *z*) is the 2D Fourier transform of (1 − 2*p*_*b*_)*p*_Forward_(*θ*, *z*). We can recognise *S* contains contributions from all scattering orders 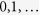 by expanding the second exponential term to 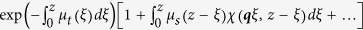 with the order 0 being the ballistic term. The reflectance of the SAA photons can be written as


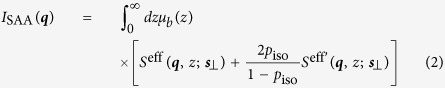


where the backscattering coefficient *μ*_*b*_ ≡ *μ*_*s*_(*z*)*p*(*π*, *z*), ***s***_⊥_ ≡ ***s***_⊥in_ + ***s***_⊥out_ with ***s***_⊥in_ and ***s***_⊥out_ being the incident and remission angles, respectively, *S*^eff^ is the spread function for an effective medium with an identical phase function and twice absorption and scattering, and *S*^eff′^ is the spread function for a second effective medium with an identical phase function and scattering whereas the absorption coefficient being modified to 2*μ*_*a*_ + 2*p*_*b*_*μ*_*s*_. The second term in equation (2) accounts for the extra contribution when the photon enters or escapes the medium taking the isotropic rather than forward scattering route whose probabilities are *p*_iso_ and 1 − *p*_iso_ 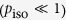, respectively (see Fig. 1a and Supplementary Materials Sec. I). The inclusion of the second term in the improved small-angle scattering approximation increases significantly the accuracy of *I*_SAA_ (see Fig. 1b). The second term is absent in the conventional small-angle approximation to radiative transfer^12^. Note the ballistic term in *S*^eff′^ should be removed to avoid double counting.

For simplicity, we will limit the discussion to a uniform semi-infinite medium and further assume *p*_Forward_(*θ*) is Gaussian hereafter. This choice is justified by first *p*_Forward_(*θ*) is dominated by large scattering structures which scatter light mainly through Fraunhofer diffraction with a Gaussian angular dependence near the forward direction and second the summation of the forward scattering phase function from many different scattering structures in a complex biological system approaches a Gaussian function according to the central limit theorem^13^. Other forms of *p*_Forward_ may be used and the Gaussian form offers a balance between simplicity and performance. The arbitrary phase function *p*(*θ*) of the scattering medium will be mapped to (see Supplementary Materials Sec. II):


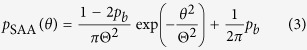


where the isotropically scattering term


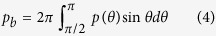


and the forward scattering angular width


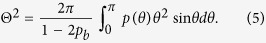


The *n*th order moment of *p*_SAA_ is given by 
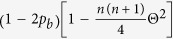
. In particular, the anisotropy factor (*n* = 1) is


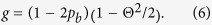


The value of *p*_*b*_ is determined by how light is backscattered into the back hemisphere whereas the backscattering coefficient *μ*_*b*_ depends on the phase function at the 180 degree which is typically different from *p*_*b*_. The probabilities for photons being forward or isotropically scattered are 1 − 2*p*_*b*_ and 2*p*_*b*_, respectively. The parameter *p*_iso_ = 2*p*_*b*_ in equation (2). The spread function (1) reduces to


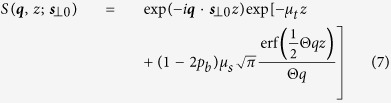


where erf is the error function. The reflectance of the SAA photons (2) incorporates contributions from *all* moments of the phase function in contrast to other approximate solutions to radiative transfer which truncate the order of the moments of the phase function^14^. The inclusion of higher order moments of the phase function is critical to accurately describe photon migration at a close source-detector separation or a high spatial frequency^6^,^15^.

The expression of *p*_SAA_ is consistent with the unified Mie and fractal model of light scattering by tissue and cells^16^,^17^,^18^. The Gaussian term captures the contribution from Mie scatterers whereas the isotropic scattering term is associated with the refractive index fluctuation of the background. The mean squared root scattering angle Θ tends to decrease with the size of Mie scatterers (large structures) in tissue and cells.

The reflectance *I*_SAA_(***q***) given in equations (2) and (7) and its inverse 2D Fourier transform 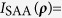

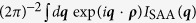
 represent the main expressions for SAA photons, governing subdiffusive light reflectance. In the typical setup with normal incidence and detection, ***s***_⊥_ = 0. When 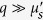 and 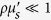, the reflectance reduces to (see Supplementary Materials Sec. III):


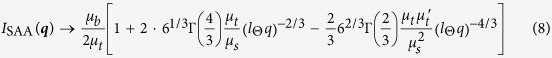


and


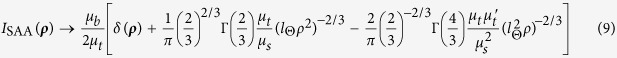


where 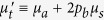, *δ*(·) is te Dirac-Delta function, and the spreading length scale *l*_Θ_ ≡ Θ/*μ*_*s*_. The first term in the bracket in equations (8) and (9) is the ballistic contribution.

The expressions for the snake and diffuse photons have been derived earlier^10^,^19^ and the more general form allowing absorption is given here:


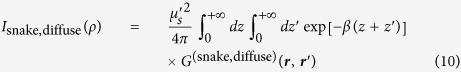


where 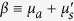, 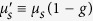 with *g* being the anisotropy factor, and *G*^(snake, diffusion)^ is the Green’s function for snake and diffusion photons, respectively. The snake Green’s function, given by 
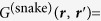
 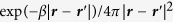, is the ballistic propagator for an isotropic source inside an isotropically scattering turbid medium. In the Fourier domain, the reflectance for snake and diffuse photons is simply:


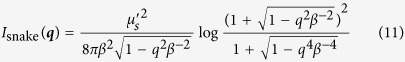


and


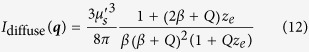


where 
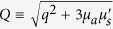
 and *z*_*e*_ is the extrapolation length dependent on the refractive index mismatch at the interface^20^.

**Figure 2:**
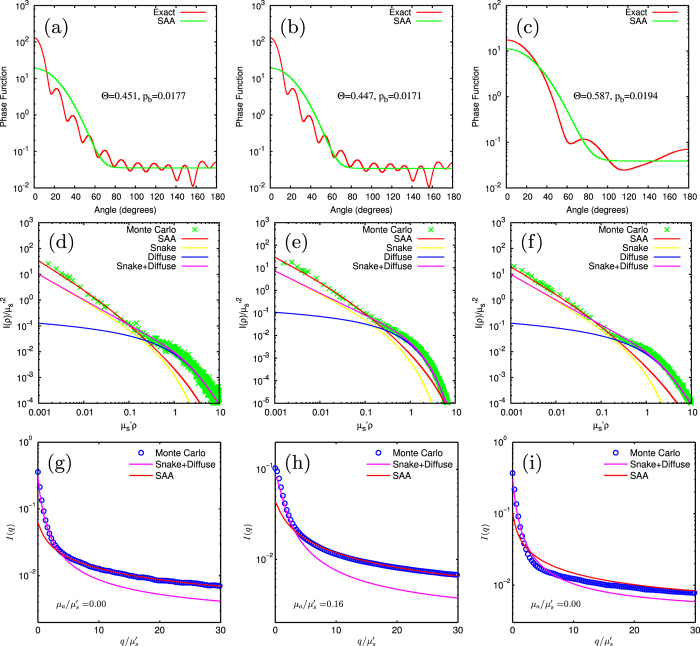
Light reflectance by SAA, snake, and diffuse photons compared with the results from Monte Carlo simulations for polystyrene sphere suspensions of diameter 1.5 *μm* ((**a**,**d**,**g**): no absorption, (**b**,**e**,**h**): 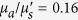; *g* = 0.92) and diameter 0.49 *μm* ((**c**,**f**,**i**): no absorption, *g* = 0.86) in water. The wavelength of the incident light is 0.515 *μm*. The top row shows the SAA phase function used to approximate the exact Mie phase function. The middle row shows the spatial profile of the reflectance at the radial position *ρ* from a normally incident collimated beam at the origin. The bottom row shows the reflectance for normally incident spatially modulated plane wave with a spatial modulation frequency ***q***. Light reflectance in (**d**–**i**) is computed directly based on the mapped SAA phase function without any free adjustable parameters.

Figure 2 compares light reflectance by SAA, snake, and diffuse photons with the results from Electric field Monte Carlo simulations^21^ in both spatial and Fourier domains for polystyrene sphere suspensions of diameter 1.5 *μm* (without and with absorption: 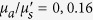) and 0.49 *μm* (no absorption) in water. The parameters for the mapped *p*_SAA_ is computed from the original phase function by equations (4) and (5). In Monte Carlo simulations, the total number of incident photons is set to 10^6^ and the refractive indices of the semi-infinite medium and the surrounding are assumed to be matched. The absorption of the scattering medium is introduced by assigning a non-zero imaginary part to the refractive index of the polystyrene particle. The mapped SAA parameters are *p*_*b*_ = 0.0177 and Θ = 0.451, *p*_*b*_ = 0.0171 and Θ = 0.447, and *p*_*b*_ = 0.0194 and Θ = 0.587, respectively (Fig. 2 top row). The SAA photons and the combined snake and diffuse photons, respectively, describes well the reflectance at short and large 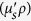 separations in the real space (Fig. 2 middle row) and at high and low 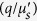 spatial frequencies in the Fourier domain (Fig. 2 bottom row).

The total light reflectance is given by the sum of contributions from both non-diffuse and diffuse photons. For the arbitrary separation and over the full spatial frequency domain, the reflectance profile can be split into low and high spatial frequency regimes specified by their respective limits, i.e.,


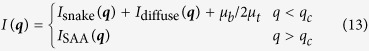


where at *q*_*c*_ ~ 2*πβ* the two limits intersect. The diffuse and non-diffuse expressions should be applied in their respective domains. The term *μ*_*b*_/2*μ*_*t*_ in the low spatial frequency expression originates from the ballistic contribution of SAA photons. This split is consistent with the observation^10^ of a universal radial profile independent of the specific form of the phase function for light backscattering at a separation larger than *β*^−1^. A matched refractive index has been assumed at the interface here. When there is an index-mismatch, the specular reflection at the entry into the semi-infinite medium can be easily taken into account by multiplying the reflectance *I*(***q***) in equation (13) with the transmission coefficient 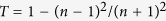 where *n* is the relative refractive index between the two media at the interface; furthermore, the SAA, snake photons and the ballistic term, *I*_SAA_, *I*_snake_ and *μ*_*b*_/2*μ*_*t*_, respectively, will be attenuated by *T* again when escaping the medium and the extrapolation length *z*_*e*_ for the diffuse photons will vary with the mismatch. It should also be pointed out that our formalism is limited to scalar photons. The polarization effect of photons can be incorporated following the recipe given in Xu and Alfano^22^,^23^.

Expression (13) provides an excellent description of light reflectance from a forward-peaked scattering medium of the anisotropy factor 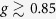 and low to moderate absorption 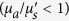. The merit of match between the model and results from Monte Carlo simulations computed over 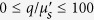 in Fourier space is shown in Table 1. Here merit of match between the model and Monte Carlo simulations is defined by


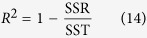


where 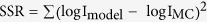 is the squared error of the model and 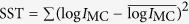 is the variation of the truth with 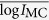 being the average of log *I*_MC_. A value of *R*^2^ = 1 means a perfect match between the model and the truth. When absorption increases 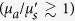 such that photons no longer completely randomise in their propagation directions or light scattering in the medium is not forward-peaked, the accuracy of equation (13) deteriorates.

**Figure 3:**
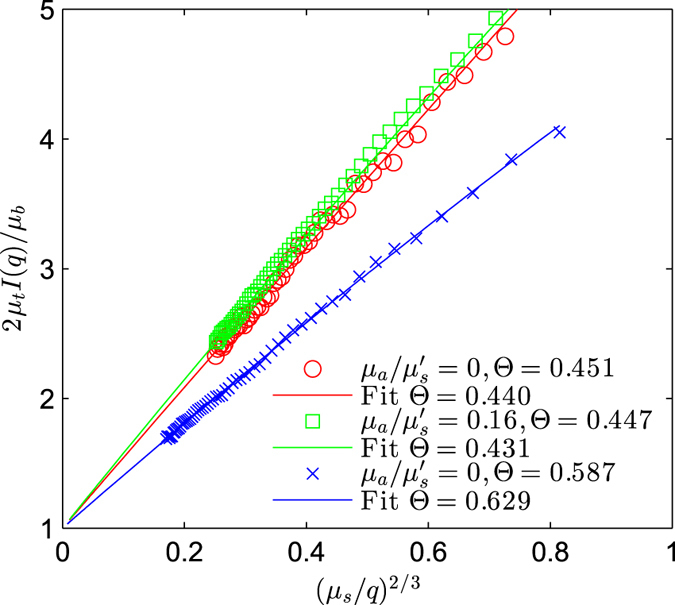
The fitting of the large *q* limit expression (8) to Monte Carlo simulations for polystyrene suspensions displayed in Fig. (2). The angular spread Θ of light scattering is determined with accuracy of 2.4%, 3.6%, and 5.6%, respectively.

The closed form light reflectance (13) and the limit forms (8,9) reveal that in addition to the well known transport mean free path *l*_*t*_ = 1/(1 − *g*)*μ*_*s*_ which governs light diffusion and the reflectance at low spatial frequency (large separation), there is a new spreading length scale *l*_Θ_ ≡ Θ/*μ*_*s*_ which characterises subdiffusive light reflectance at high spatial frequency (close separation) solely determined by the angular width of the forward-peaked component of the phase function. The two length scales are further inherently connected via equation (6) for a forward-peaked scattering medium, satisfying approximately 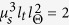. Light reflectance at high frequency (close separation) provides a convenient measure of *l*_Θ_ via equations (8) and (9). Figure 3 shows the fitting of equation (8) to Monte Carlo simulations for polystyrene suspensions displayed in Fig. (2). The angular spread Θ of light scattering is determined with accuracy of 2.4%, 3.6%, and 5.6%, respectively.

Moreover, the complete set of the optical properties of the turbid medium can be determined accurately by fitting to light reflectance at both low and high spatial frequencies. The set of parameters includes the mapped SAA phase function (3) completely defined by the angular spread Θ of scattering and the isotropic scattering background *p*_*b*_ for an arbitrary phase function of the scattering medium, the backscattering coefficient *μ*_*b*_, the scattering coefficient *μ*_*s*_, the absorption coefficient *μ*_*a*_, and the anisotropy factor *g*. As one example, the fitted parameters from light reflectance simulated by Monte Carlo simulations for the polystyrene sphere suspension (*d* = 1.50 *μm*, 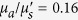) is shown in Table 2. The values of *μ*_*b*_/*μ*_*s*_, *μ*_*a*_/*μ*_*s*_ and Θ/*μ*_*s*_ are first fitted from subdiffusive light reflectance and then the full set of optical parameters are fitted against both subdiffusive and diffusive light reflectance profile with least square curve fitting when fixing *μ*_*b*_/*μ*_*s*_ and setting *g* = (1 − 2*p*_*b*_)(1 − Θ^2^/2). The complete set of optical parameters determined from light reflectance agree well with their theoretical values. In particular, the accuracy of the extracted scattering properties and phase function parameters (*μ*_*s*_, *g* and Θ) is remarkable which can be partly attributed to the inherent constraint (6) between *g* and Θ, which characterises diffusive and subdiffusive light reflectance respectively, imposed by a forward-peaked scattering medium.

## Summary

In summary, we have presented here an analytical model describing light reflectance at an arbitrary source-detector separation from forward-peaked scattering media and its application in diagnosis of the phase function of such media. The model incorporates the small-angle scattering approximation to radiative transfer for sub-diffusive light reflectance. This analytical model exhibits excellent performance over the whole spatial length scales when light absorption is weak to moderate. The application of the model in accurate determination of the optical properties and the phase function of a turbid medium from the profile of subdiffusive and diffusive light reflectance has also been successfully demonstrated. The phase function of a scattering medium carries the ultimate information about the morphology and optical properties of the individual scatterers which can be probed remotely. The diagnosis of the phase function, furthermore, can be used to predict light propagation and detect minute structural alterations or heterogeneities inside a random medium. With the recent development such as the spatially modulated illumination^24^, both subdiffusive and diffusive light reflectance over a wide field can be easily assessed. This enables a rapid quantification of the complete set of the optical properties, including the phase function, of the scattering media using the proposed analytical model. Such noninvasive approach will hence find important applications in biomedical optics and remote sensing, in general, for diagnosis of tissue and other forward-peaked scattering media from light reflectance measurements.

## Additional Information

**How to cite this article**: Xu, M. Diagnosis of the phase function of random media from light reflectance. *Sci. Rep.* **6**, 22535; doi: 10.1038/srep22535 (2016).

## Supplementary Material

Supplementary Information

## Figures and Tables

**Table 1:** Merit of match between the model and Monte Carlo simulations for polystyrene sphere suspensions (diameter *d* and absorption *μ*_*a*_).

	*d* = 1.50 *μm*, *μ*_*a*_ = 0	*d* = 1.50 *μm*, 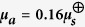	*d* = 0.49 *μm*, *μ*_*a*_ = 0
*R*^2^	0.9915	0.9877	0.9668

The value of *R*^2^ = 1 means a perfect match between the model and the truth.

**Table 2:** The fitted parameters from light reflectance simulated by Monte Carlo simulations for the polystyrene sphere suspension (*d* = 1.50 *μm*, 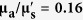 ) compared to their theoretical values.

	*μ*_*s*_(cm^−1^)	*μ*_*a*_(cm^−1^)	*g*	*p*_*b*_	Θ	*μ*_*b*_(cm^−1^)
Theoretical	1.000	0.0130	0.921	0.0173	0.447	0.00388
Fitted	1.019	0.0116	0.915	0.0204	0.431	0.00331
