# Supplementary material for: Diagnosis of the phase function of random media from light reflectance
Source: Sci Rep. 2016 Mar 3;6:22535. doi: 10.1038/srep22535 (PMC4776107; doi:10.1038/srep22535)
Supplement: Supplementary Information [file srep22535-s1.pdf]

Diagnosis of the phase function of random media from light reflectance

Min Xu
Physics Department, Fairfield University, CT 06824, USA

1 The correction to light reflectance by the second effective medium

Backscattering of the first order non-diffuse photons encounter multiple small-angle scattering and one single large angle scattering at which light bounces back. The photon may take the isotropic vs forward scattering route with the probability of $p_{\rm iso} = 2p_b$ and $1 - p_{\rm iso}$, respectively, at the first or last scattering event. The correction to light reflectance corresponds to the photons which experiences the single isotropic scattering at the first (or last) scattering event and can be written as

$$2\int_0^\infty dz\mu_b(z)p_{\rm iso}\exp\left[-\int_0^z \mu_a(\xi)d\xi - \int_0^z 2p_b(\xi)\mu_s(\xi)d\xi\right]S(\boldsymbol{q}, z; \boldsymbol{s}_\perp)$$

which is identical to

$$\int_0^\infty dz\mu_b(z)2p_{\rm iso}S^{\rm eff'}(\boldsymbol{q}, z; \boldsymbol{s}_\perp).$$

Noting that the term $\int_0^\infty dz\mu_b(z)S^{\rm eff}(\boldsymbol{q}, z; \boldsymbol{s}_\perp)$ associates with the first (or last) scattering event takes the forward scattering route with a probability $(1 - p_{\rm iso})$, the correction to $S^{\rm eff}(\boldsymbol{q}, z; \boldsymbol{s}_\perp)$ is hence

$$\frac{2p_{\rm iso}}{1 - p_{\rm iso}}S^{\rm eff'}(\boldsymbol{q}, z; \boldsymbol{s}_\perp).$$

2 The mapping of arbitrary phase function $p(\theta)$ to $p_{\rm SAA}(\theta)$

The SAA phase function contains a Gaussian forward-scattering peak and an isotropic component which takes the form of Eq. (3) under the normalization requirement that

$$2\pi\int_0^\pi p_{\rm SAA}(\theta)\sin\theta d\theta = 2\int_0^\infty \frac{1-2p_b}{\Theta^2}\exp\left(-\frac{\theta^2}{\Theta^2}\right)\theta d\theta + \int_0^\pi p_b\sin\theta d\theta = 1.$$

An arbitrary phase function $p(\theta)$ with the normalization $2\pi\int_0^\pi p(\theta)\sin\theta d\theta = 1$ is mapped to the SAA phase function. The spread of the scattering angles is matched over the full 4π space and the backscattering component is matched using the backward hemisphere. That is,

$$2\pi\int_0^\pi p(\theta)\theta^2\sin\theta d\theta = 2\pi\int_0^\pi p_{\rm SAA}(\theta)\theta^2\sin\theta d\theta \simeq 2\int_0^\infty \frac{1-2p_b}{\Theta^2}\exp\left(-\frac{\theta^2}{\Theta^2}\right)\theta^3 d\theta = (1-2p_b)\Theta^2$$

and

$$2\pi\int_{\pi/2}^\pi p(\theta)\sin\theta d\theta = 2\pi\int_{\pi/2}^\pi p_{\rm SAA}(\theta)\sin\theta d\theta \simeq \int_{\pi/2}^\pi p_b\sin\theta d\theta = p_b.$$

This proves the mapping equations (4) and (5).

3 The high frequency (close separation) limit of $I_{\rm SAA}$

The spread function S can be approximated by:

$$S(\boldsymbol{q}, z) \simeq \begin{cases} \exp\left[-\mu_t' z - \frac{1}{12}(1-2p_b)\mu_s\Theta^2 q^2 z^3\right] & \Theta q z < 2 \\ \exp\left[-\mu_t z + (1-2p_b)\mu_s\frac{\sqrt{\pi}}{\Theta q}\right] & \Theta q z > 2 \end{cases}$$

noting ${\rm erf}(x) = \frac{2}{\sqrt{\pi}}(1 - \frac{x^2}{3})x$ when x is small and $\mu_t' \equiv \mu_a + 2p_b\mu_s$. As the regime where $\Theta q z < 2$ is satisfied coincides with the validity regime of the SAA, we will relax this restriction and rewrite the SAA spread function as

$$S(\boldsymbol{q}, z) \quad = \quad \exp\left[-\mu_t' z - \frac{1}{12}(1-2p_b)\mu_s\Theta^2 q^2 z^3\right] \tag{1}$$

over the whole $\boldsymbol{q}$ and z domain. The corresponding approximate spread function in the spatial space is given by

$$S(\boldsymbol{\rho}, z) = \frac{3}{\pi(1-2p_b)\mu_s\Theta^2 z^3} \exp\left[-\mu_t' z - \frac{3\rho^2}{(1-2p_b)\mu_s\Theta^2 z^3}\right].$$

It is clear that the approximate form of the spread function does not include the ballistic term.

In the limit of high frequency,

$$\begin{aligned}
\int_0^\infty S(\boldsymbol{q}, z)dz &= \int_0^\infty \exp\left[-\mu_t' z - \frac{1}{12}(1-2p_b)\mu_s\Theta^2 q^2 z^3\right] dz \\
&\simeq \int_0^\infty \exp\left[-\frac{1}{12}(1-2p_b)\mu_s\Theta^2 q^2 z^3\right] dz - \mu_t' \int_0^\infty z \exp\left[-\frac{1}{12}(1-2p_b)\mu_s\Theta^2 q^2 z^3\right] dz \\
&= \Gamma(\frac{4}{3}) \left[\frac{1}{12}(1-2p_b)\mu_s\Theta^2 q^2\right]^{-1/3} - \mu_t' \frac{1}{3}\Gamma(\frac{2}{3}) \left[\frac{1}{12}(1-2p_b)\mu_s\Theta^2 q^2\right]^{-2/3}.
\end{aligned}$$

and correspondingly

$$\int_0^\infty S(\boldsymbol{\rho}, z)dz = \frac{1}{3\pi}\Gamma(\frac{2}{3}) \left[\frac{1}{3}(1-2p_b)\mu_s\Theta^2\right]^{-1/3} \rho^{-4/3} - \mu_t' \frac{1}{\pi}\Gamma(\frac{4}{3}) \left[\frac{1}{3}(1-2p_b)\mu_s\Theta^2\right]^{-2/3} \rho^{-2/3}$$

for small ρ.

Light reflectance at the high frequency (short separation) limit is then given by

$$\begin{aligned}
I_{\mathrm{SAA}}(\boldsymbol{q}) &\to \frac{\mu_b}{2\mu_t} + \mu_b\Gamma(\frac{4}{3}) \left(1 + 2^{1/3}\frac{4p_b}{1-2p_b}\right) \left[\frac{1}{6}(1-2p_b)\mu_s\Theta^2 q^2\right]^{-1/3} \\
&\quad -\mu_b\mu_t' \frac{1}{3}\Gamma(\frac{2}{3}) \left(1 + 2^{2/3}\frac{4p_b}{1-2p_b}\right) \left[\frac{1}{6}(1-2p_b)\mu_s\Theta^2 q^2\right]^{-2/3} \\
&\simeq \frac{\mu_b}{2\mu_t}\left[1 + 2\cdot 6^{1/3}\Gamma(\frac{4}{3})\frac{\mu_t}{\mu_s}\left(\frac{\mu_s}{\Theta q}\right)^{2/3} - \frac{2}{3}6^{2/3}\Gamma(\frac{2}{3})\frac{\mu_t\mu_t'}{\mu_s^2}\left(\frac{\mu_s}{\Theta q}\right)^{4/3}\right] \\
&= \frac{\mu_b}{2\mu_s}\left[\frac{\mu_s}{\mu_t} + 2\cdot 6^{1/3}\Gamma(\frac{4}{3})\left(\frac{\mu_s}{\Theta q}\right)^{2/3} - \frac{2}{3}6^{2/3}\Gamma(\frac{2}{3})\frac{\mu_t'}{\mu_s}\left(\frac{\mu_s}{\Theta q}\right)^{4/3}\right]
\end{aligned}$$

and

$$\begin{aligned}
I_{\mathrm{SAA}}(\boldsymbol{\rho}) &\to \frac{\mu_b}{2\mu_t}\delta(\boldsymbol{\rho}) + \mu_b\frac{1}{3\pi}\Gamma\left(\frac{2}{3}\right)\left(1 + 2^{1/3}\frac{4p_b}{1-2p_b}\right)\left[\frac{2}{3}(1-2p_b)\mu_s\Theta^2\right]^{-1/3}\rho^{-4/3} \\
&\quad -\mu_t'\frac{1}{\pi}\Gamma(\frac{4}{3})\left(1 + 2^{2/3}\frac{4p_b}{1-2p_b}\right)\left[\frac{2}{3}(1-2p_b)\mu_s\Theta^2\right]^{-2/3}\rho^{-2/3} \\
&\simeq \frac{\mu_b}{2\mu_t}\left[\delta(\boldsymbol{\rho}) + \frac{1}{\pi}\left(\frac{2}{3}\right)^{2/3}\Gamma\left(\frac{2}{3}\right)\frac{\mu_t}{\mu_s}\left(\frac{\mu_s}{\Theta\rho^2}\right)^{2/3} - \frac{2}{\pi}\left(\frac{2}{3}\right)^{-2/3}\Gamma(\frac{4}{3})\frac{\mu_t\mu_t'}{\mu_s^2}\left(\frac{\mu_s^2}{\Theta^2\rho}\right)^{2/3}\right] \\
&= \frac{\mu_b}{2\mu_s}\left[\frac{\mu_s}{\mu_t}\delta(\boldsymbol{\rho}) + \frac{1}{\pi}\left(\frac{2}{3}\right)^{2/3}\Gamma\left(\frac{2}{3}\right)\left(\frac{\mu_s}{\Theta\rho^2}\right)^{2/3} - \frac{2}{\pi}\left(\frac{2}{3}\right)^{-2/3}\Gamma(\frac{4}{3})\frac{\mu_t'}{\mu_s}\left(\frac{\mu_s^2}{\Theta^2\rho}\right)^{2/3}\right].
\end{aligned}$$
